# Development of the Arabic Health Measures database: a bibliometric analysis of Arabic health-related measures

**DOI:** 10.1186/s12961-022-00890-7

**Published:** 2022-08-09

**Authors:** Nada M. Albawardi, Quratulain Shaikh, Wejdan Alahaideb, Maryam Alamasi, Doaa Aljasser, Lama Alrasheed, Sultanah H. Alsulaiman, Abdullah F. Alghannam

**Affiliations:** 1 Epidemiology and Biostatistics Section, Health Sciences Research Center, Princess Nourah Bint Abdulrahman University, Riyadh, Saudi Arabia; 2 Epidemiology Department, Indus Hospital Research Center, The Indus Hospital, Karachi, Pakistan; 3 Lifestyle and Health Research Center, Health Sciences Research Center, Princess Nourah Bint Abdulrahman University, Riyadh, Saudi Arabia

**Keywords:** Arabic, Measures, Tools, Surveys, Translation

## Abstract

**Background:**

To develop an open-access database of Arabic health measures intended for use by researchers and healthcare providers, along with a bibliometric analysis of the measures included in the database.

**Methods:**

A search was conducted up to 31 December 2021 in PubMed, Embase, CINAHL, SAGE, Springer and Elsevier for published articles or abstracts with keywords “Arabic” AND “translation”, “adaptation” OR “validation”. Information on the measure and the methodology used in the study was then entered into a database. An open-access platform was developed to allow users to search for measures according to their needs. A bibliometric analysis of the articles and measures was then conducted.

**Results:**

A total of 894 publications met the inclusion criteria. The articles discussed 716 measures that were developed using participants from at least 38 countries. The number of measures for adults was five times that for children. Mental health was the most frequent construct assessed (11.5%), followed by “function/disability” measures (10.6%). The majority of measures (54%) required 5 minutes or less to complete. Approximately 17% of the tools were available directly from the article. Saudi Arabia and Lebanon had the greatest number of publications, with 217 (23%) and 114 (12%), respectively. The majority of the publications included reporting of the validation and reliability of the instruments (64% and 56%, respectively).

**Conclusions:**

There is a paucity of research on the quantity and quality of Arabic health measures. Similar to previous reviews, we found the number of publications on Arabic measures to be limited in comparison to those in English; however, it is encouraging that the number of publications appears to have increased steadily over the past decade. While we found the majority of publications reported on psychometric testing, we are unable to comment on the quality of the methodology used, and further investigation into this area is recommended. As the Arabic Health Measures database will facilitate the search for health instruments that have published data on their development, this will increase their visibility and use in research and clinical settings.

**Supplementary Information:**

The online version contains supplementary material available at 10.1186/s12961-022-00890-7.

## Background

Valid and reliable measurements have long been a pillar of research methodology. With the use of standardized measurement tools rapidly extending from the realm of research to the healthcare setting, there is increasing demand for rapid access to these instruments. Health measurements—also referred to as tools or instruments—are defined as a “tool for data collection [that] serves as the mechanism for gathering data about the concept or attribute(s) of interest’’ [1]. Health measurements are used to collect data on a variety of constructs, ranging from physical functioning to psychosocial well-being and are used for screening, diagnosis and measuring outcomes that may be self-reported or conducted by the researcher or healthcare provider. These instruments allow healthcare workers to analyse patients at various stages of care, as well as providing valuable information that may be used to develop best practice strategies. At the institutional level, instruments that evaluate important performance indicators such as patient safety and compliance to standards of care, allow objective measurement of healthcare facility outcomes and benchmarking between organizations at the local and international levels.

As multinational and multicultural research projects increase, the need to provide valid health measures for use in languages other than the source language has grown rapidly. The validity and reliability of such measures is paramount to their use. Most instruments including questionnaires, checklists, rating scales, interviews and protocols rely on written or spoken language in their delivery. Similar to most medical literature, questionnaires were originally developed in English-speaking countries [2]. As language can greatly affect the psychometric properties of an instrument, availability of validated versions of such instruments in the native language of the user is key to promoting their use and ensuring valid outcomes in settings where English is not the main medium of communication. The translation procedure must take into consideration not only semantic equivalence, in which words and sentence structure in the translated text express the same meaning as the source language, but also conceptual equivalence which ensures that the concept being measured is the same, regardless of whether the wording is different [3]. Directly translating an instrument from the source language without concern for the linguistic and cultural subtleties that influence the intended meaning of the question, sometimes referred to as adaptation of the instrument [4], is likely to affect its validity and reliability. When instruments are used across varying cultures, normative equivalence must also be considered. This ensures the ability of the translated text to address variation in social norms such as issues specific to religion or health beliefs [3]. In this case, components of the instrument may be modified or altered (independent of changes made as a result of the translation) to make them suitable for use in the target population. This process of adaptation ensures the semantic, conceptual and normative equivalence of the instrument [5].

The number and scope of health measures has increased significantly in the past decade and covers many languages, cultures and regions [6]. Over the past few decades, medical instrument databases have been developed to improve selection, access and appraisal of instruments. These databases either are repositories or provide links to instruments covering a range of topics including social sciences [7], rehabilitation [8], health literacy [9] and care coordination [10]. While Arabic is ranked as the fourth most frequently spoken language in the world, with the number of people speaking Arabic as a first language estimated at 315 million and spread across 58 countries [11], no open-access database of Arabic health measures was identified. We believe that a database of Arabic measures would increase the visibility of previously developed instruments and decrease the effort involved in searching for appropriate tools. This would likely increase their use and facilitate greater standardization of measurement procedures. A database would also highlight areas of need and may prompt greater interest in the development of valid health measures. The aim of this project was to develop an open-access database of Arabic health measures intended for use by researchers or healthcare providers that would allow users to search for instruments according to the construct required and would provide links to the articles describing their development.

Our objectives were as follows:

1. Perform a thorough literature search on health-related measurement tools that were originally developed in Arabic or translated, validated and/or adapted from another language to the Arabic language.
2. Design an electronic database which allows users to search for health-related measurement tools that were originally developed in Arabic or were translated to the Arabic language according to keywords, the construct required and other characteristics of the instrument.
3. Share the designed database by virtue of an open-access website in order to provide a common platform for access and dissemination of these measurement tools, with due respect to copyright issues.
4. Conduct a bibliometric analysis of available Arabic health measures identified through a comprehensive literature search up to December 2021.

## Methods

### Ethical considerations

This project was granted exempt status from the ethical review board at Princess Nourah bint Abdulrahman University (institutional review board [IRB] log 17-0177).

### Literature search

The literature search was conducted from January 1985 to December 2021. In the initial phase, we searched in PubMed, Embase, CINAHL, SAGE, Springer and Elsevier for published articles or abstracts with keywords “Arabic” AND “translation”, “adaptation” OR “validation”. Boolean search terms were used to ensure a comprehensive search of available literature. Subsequently, a second search phase was conducted as follows:

- specifically looking for similar instrument development articles by prominent authors identified in the initially retrieved articles;
- searching for the *development* of a measure from articles that were excluded because they described only the *use* of the measure; and
- hand search of reference lists.

To ensure proper utility and consistency, the database will be updated biannually using the aforementioned search strategy.

### Study selection criteria

Study selection criteria included any article, published in the English language, reporting on a health-related tool or measurement which had been either (1) translated or adapted from another language to Arabic, or (2) originally developed in the Arabic language.

The tool was included in the database if its development, translation, adaptation and/or psychometric evaluation were described in the article. Studies reporting the development of such measures without any report on the translation process and/or psychometric properties were excluded.

All retrieved articles were archived in an EndNote reference management software file (EndNote X9, Clarivate Analytics, USA). In the first round of data cleaning, a junior member of the team reviewed the titles and abstracts to determine whether the inclusion criteria were met; if this was unclear, the full text was obtained and reviewed. Excluded studies were reviewed by a senior member of the team to ensure irrelevance to the aim and were archived in separate folders within the same EndNote file, along with the reason for exclusion. Any ambiguous article was reviewed by a second senior member of the team, and a consensus decision for exclusion was reached by the two senior members.

### Data extraction

Data were extracted for each included article and entered into a password-protected Microsoft Access database pre-designed for the project, and now the data are stored in a MySQL database. Information on the measure discussed in the article was then entered by reviewing the information in the article and, when necessary, searching for information on the original measure (Additional file 1). In order to adhere to copyright laws, links to the measurement tool and /or the full text of the article were included if they were available as open access; otherwise only the URL to the article abstract was provided. If the measure was not available as open access, the corresponding author’s email was entered into the database in order to facilitate access to the measure. A team of four junior epidemiologists extracted the relevant information on the pre-designed database after a pilot entry of 20 articles. Issues identified in the pilot were revisited by senior members of the team and resolved in order for the actual data extraction to work smoothly.

### Development of the Arabic Health Measures (AHM) website

The database is accessible through a website which will serve as a unique and comprehensive hub for access to Arabic health-related measures not only within the country or region but globally as well (Additional file 2). Development of the website was guided/inspired by the following:

- a guideline for website design by the United States Department of Health and Human Services [12];
- prominent English websites with healthcare measures databases [7–10]; and
- face-to-face interviews with potential users—conducted with four professionals with a background in health and research, with the objective of gathering information on the user’s needs and preferences in using such a database.

## Results

**Fig. 1:**
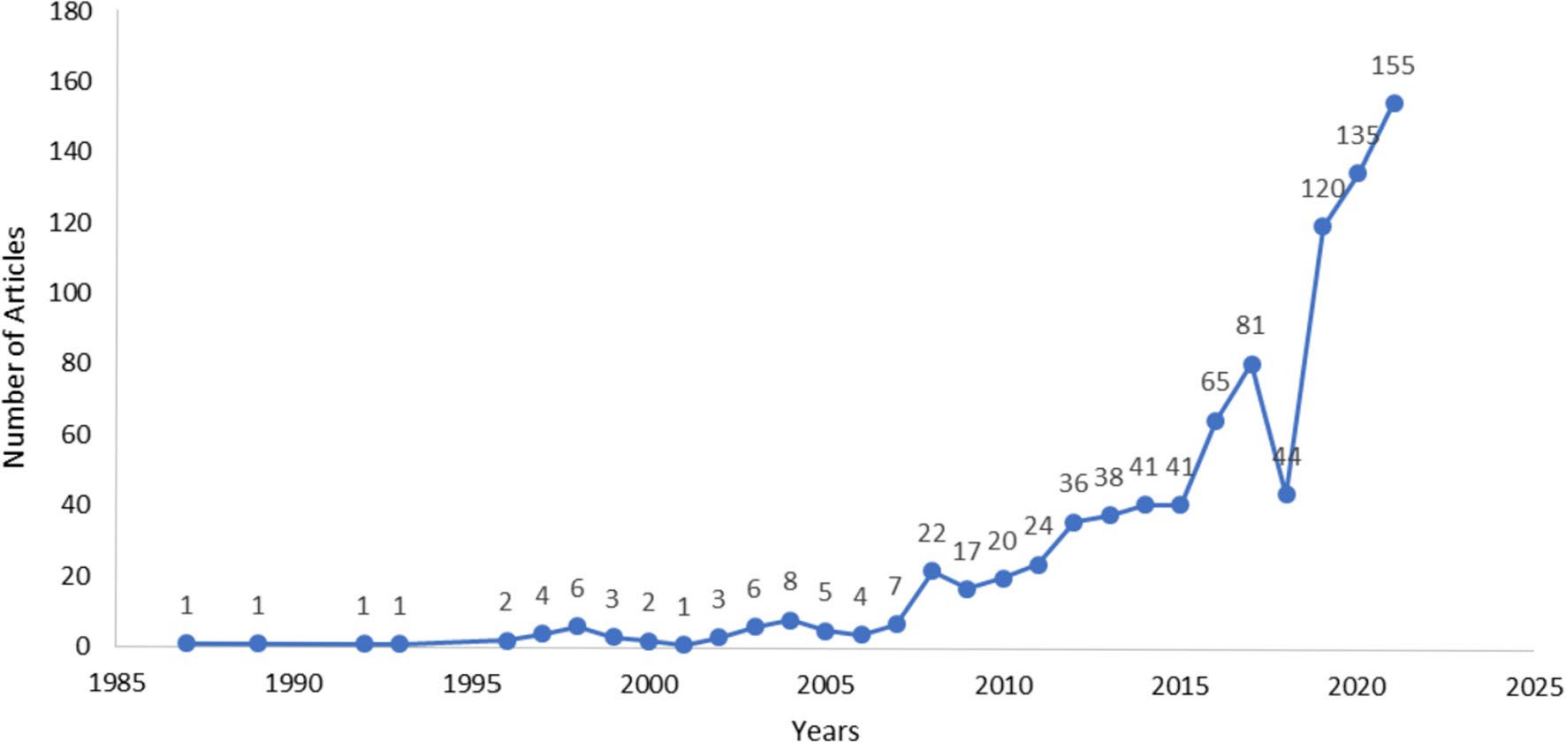
Number of publications on translation and/or psychometric testing of Arabic health measures per year

**Fig. 2:**
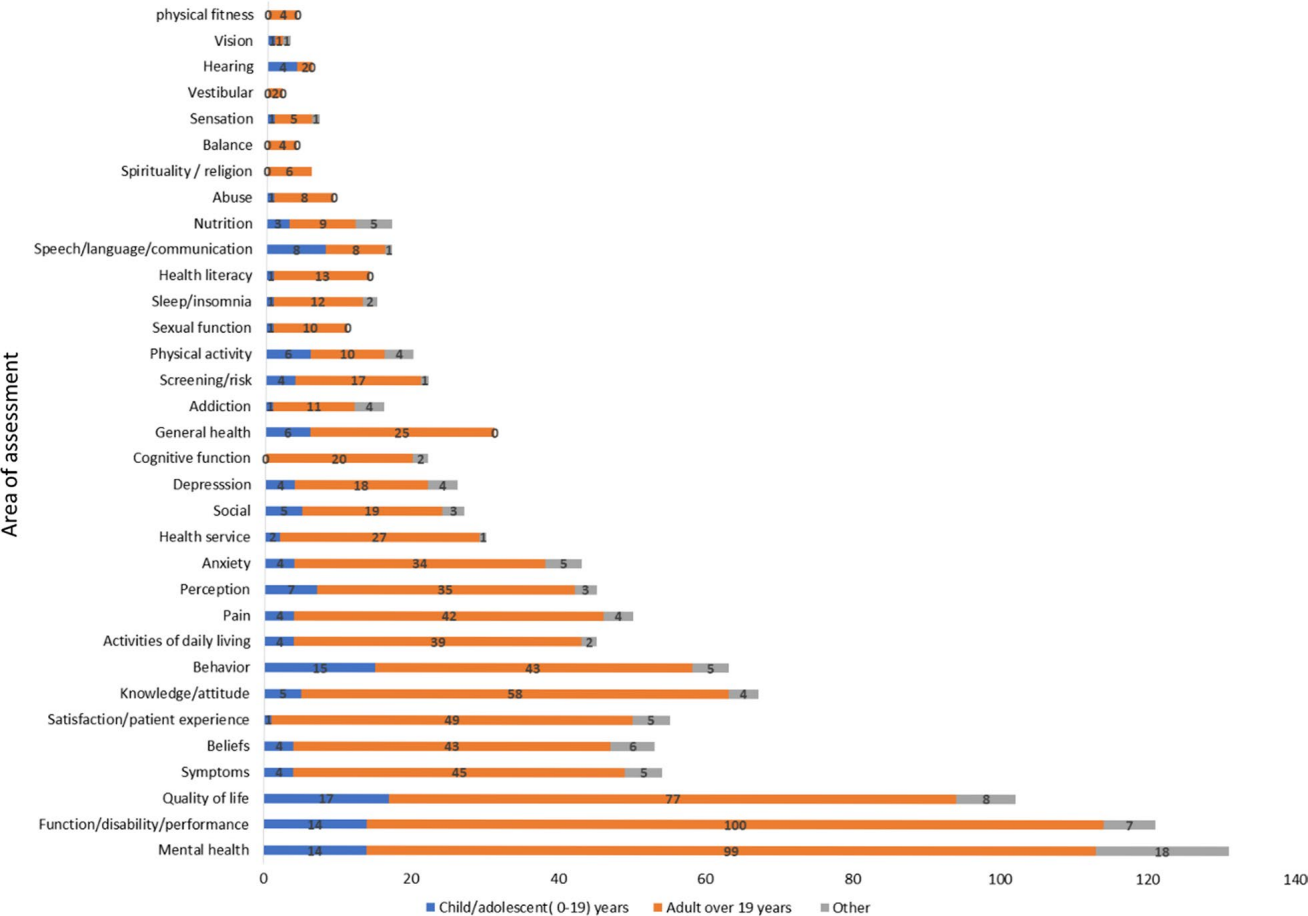
Number of Arabic health measures according to area of assessment and age category

**Fig. 3:**
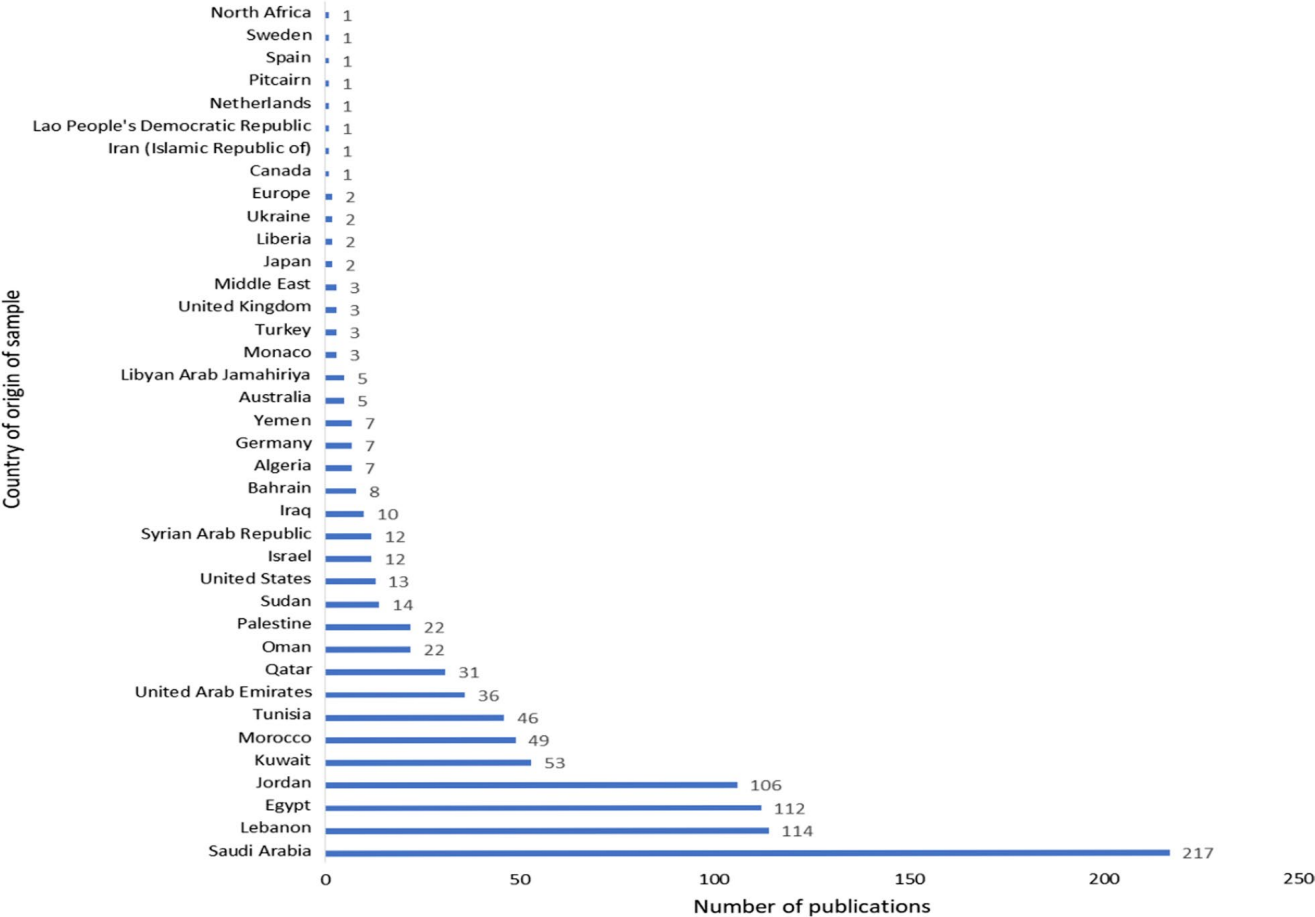
Number of publications in the AHM database according to country of origin of sample (*n* = 1138)

**Fig. 4:**
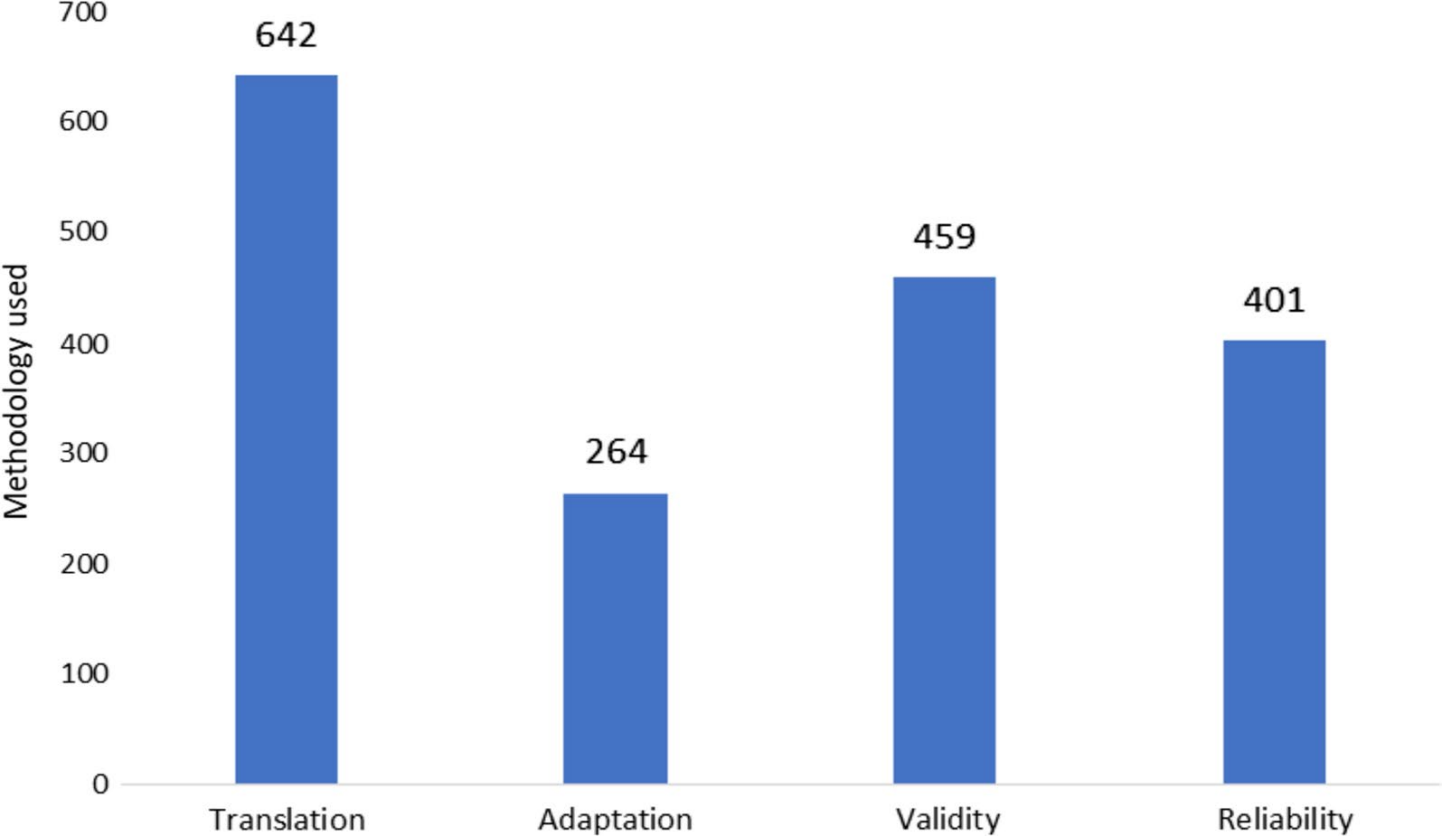
Methodology used in publications on the development of Arabic health measures (*n* = 4724)

The AHM database was developed by the Health Sciences Research Center at Princess Nourah bint Abdulrahman University and launched in November 2018 [13]. It is a product of a comprehensive literature search conducted to include publications from January 1985 to December 2021. The final number of publications included in the original database was 894 (843 [94.3%] published articles, 43 [4.8%] published abstracts, seven [0.8%] theses, and one unpublished manuscript), ranging in publication year from 1987 to 2021 (Fig. 1). These articles described the development of 716 measures. The greatest numbers of instruments were found under the constructs of “mental health” (11.5%), “function/disability/performance” (10.6%) and “quality of life” (9%) (Fig. 2). As exhibited in Fig. 2, the availability of adult instruments was markedly greater in comparison to measures for children. In total, nearly five times as many adult instruments were identified as children’s instruments (895 vs 142, respectively). In describing the access to the measure, only 121 (16%) of the 716 Arabic tools were available within the article (categorized as “free”), while the majority of measures (82%) did not have means for accessing the measure documented in the article and were thus categorized as “contact author” (Table 1). The majority of tools (54%) reported the time to complete the measure as “less than 5 minutes”, followed by 249 (41%) measures taking “6 to 30 minutes” to be completed by the respondent (Table 1). The instruments were developed using participants from over 38 countries. The country of origin of the participants was most frequently Saudi Arabia, followed by Lebanon, Egypt and Jordan (217 [23%], 114 [12%], 112 [12%], 106 [11%] publications, respectively) (Fig. 3). The methodology of the majority of publications included validity testing (64%), followed by tests of reliability (56%). Translation was conducted in 89.6% of the studies, while only 36.8% were on adaptation of the measure (Fig. 4).

**Table 1:** Description of measures in the AHM database (*n* = 716)

Description	No. (%)
*Age category*	
Child (0–19 years)	85 (12)
Adult (over 19)	503 (70.5)
Other (combination of adult/child)	58 (8)
Not defined	68 (9)
*Availability of measure*	
Free	121 (16)
Pay	16 (2)
Contact author^a^	600 (82)
*Time to complete measure*	
Less than 5 minutes	326 (54)
6–30 minutes	249 (41)
31–60 minutes	24 (4)
Over 60 minutes	3 (1)
*Training required*	
Required	27 (5)
Not required	585 (95)

^a^If the article does not indicate where the Arabic version of the instrument can be accessed

## Discussion

With the use of health surveys rapidly extending from the realm of research to clinical, academic and commercial settings, the demand for valid and reliable measures is increasing. The comparability of health survey data across varying populations is also vital and has been challenging in part due to the lack of standardization of health instruments and variation in survey methodology [6]. As the Arabic language includes various dialects and culturally specific idioms [11], this adds to the challenge of producing standardized and validated instruments in the Arabic language. Instruments may also perform differently in different contexts, age groups and health conditions and thus require validation in different populations [3, 4, 14].

There is a paucity of research on the quantity and quality of Arabic health measures. Only two systematic reviews were identified that evaluated existing Arabic health measures. A systematic review on Arabic generic health-related quality-of-life measures by Al Sayah (2012) reported on 20 studies which included six measures and found moderate- to good-quality cross-cultural adaptations; however, evaluation of measurement properties was limited due to deficient evidence [15]. Fasfous et al. [16] conducted a review to evaluate the quality of studies involving the use of neuropsychological assessments of Arabic-speaking individuals. He reported on 384 studies applying 117 instruments and found that nearly half of the publications did not use cognitive tests that were “developed, translated, adapted, or standardized according to international guidelines of psychological measurement” [16]. Reviews conducted on English health measures of varying constructs have reported similar flaws in methodological quality [14, 17–19]. Furthermore, after excluding intelligence and cognitive screens, Fasfous et al. found that the three most frequently used tests—the Trail Making Test, Wechsler Memory Scale and Wisconsin Card Sorting Test—had no reporting on their validity for Arab individuals [16]. Fasfous et al. [16] also reported that while 57 tools referenced norming efforts, they were sometimes inaccessible. Similarly, our review found less than 10% of the health measures identified in our search were available directly online (Table 1), posing an additional obstacle to access to existing Arabic health measures.

Our review found Saudi Arabia and Lebanon to have the highest rate of publications reporting on the development of measures, with 217 and 114, respectively (Fig. 3). Interestingly, however, while Fasfous et al. (2017) found that these same countries had the greatest number of publications on the use of neuropsychological measures, they were ranked the lowest in reporting the validation and/ or norming of instruments [16]. Our results had a high percentage of publications reporting on validity and reliability (Fig. 4); however, as this review does not include a quality check of the methodology, it is not clear how many Arabic health measures in the AHM database meet international guidelines for psychometric testing and norming.

Similar to previous reviews [15, 16], we found the number of publications on Arabic measures to be limited in comparison to those in English [7–10]. This is likely due to several factors, including the limitation of our search to major English databases and literature published in the English language. It is also likely that more studies may be found in local journals and non-indexed or non-peer-reviewed journals. In addition, some measures are translated as part of a research project and may not have publications on their methodology, or their publications may provide limited information on their methodology, thus excluding them from our review. Finally, some translation or validation studies may never be published or may be part of the grey literature from academic institutions or working groups. Nonetheless, it is encouraging that the number of publications appears to have steadily increased over the past decade (Fig. 1) [16], indicating the importance of translation/validation studies for developing health measures that accurately evaluate an outcome being measured.

This review was limited by the inclusion of only major English databases. A search in local databases was thought to be challenging, as it would include over 25 countries, some of which have limited internet access to their journals. We also believe that not all relevant studies may have been identified, as the translation or psychometric testing of measures may have been part of a larger project and thus not identified by the keywords used.

## Conclusion

The AHM database was developed to increase the visibility and access to currently available Arabic measures while also providing a summary of the instruments’ characteristics and the studies conducted on their development. The database is intended to be used as a resource to enable researchers and healthcare providers to identify their required measures and assess the appropriateness and quality of the instruments. We believe that the information provided in the database will concomitantly highlight the varying methodologies used during the instrument development process and inspire researchers to follow standard psychometric testing protocols, which may help in standardizing psychometric testing and facilitate the use of health measures in several areas at the health system level.

### Strengths and limitations

- This article describes the development of the first open-access database of Arabic health measures.
- Only measures with publications on their development were included.
- Analysis of the characteristics of the measures identified in Arabic is presented.
- Only English databases were included, which may have limited the number of measures identified.
- The quality of methodology used to develop the measures was not assessed.

## Supplementary Information

**Additional file 1.** Article data fields. Measurement data fields.

**Additional file 2.** Use case diagram.

## Data Availability

The datasets used and/or analysed during the current study are available from the corresponding author on reasonable request.
