# Supplementary material for: Development of the Arabic Health Measures database: a bibliometric analysis of Arabic health-related measures
Source: Health Res Policy Syst. 2022 Aug 9;20:87. doi: 10.1186/s12961-022-00890-7 (PMC9361637; doi:10.1186/s12961-022-00890-7)
Supplement: Supplementary file 1 — Additional file 1. Article data fields. Measurement data fields. [file 12961_2022_890_MOESM1_ESM.docx]

| **Appendix A**  **Article Data Fields** | |
| --- | --- |
| **Data fields for article** | **Entry options** |
| DOI | If available |
| Article URL |  |
| Article title | Full title |
| First Author | Last name, first initial . middle initial (if available).  e.g. Alghwiri, A. A. |
| Journal Name | Full or abbreviated |
| Type | Published article |
|  | Published abstract |
|  | Thesis |
| Publication Year |  |
| Full Abstract | Enter full abstract |
| Full Citation | Enter in Vancouver Style e.g.  Halpern SD, Ubel PA, Caplan AL. Solid-organ  transplantation in HIV-infected patients. N Engl J Med. 2002;347(7):284-7. |
| Is full text available? | Yes |
|  | No |

| **Data fields for study**  **summary** | **Entry options** |
| --- | --- |
| Study methodology | See drop down list in database (may choose more than one) |
| Sample size |  |
| Sample condition | e.g. healthy or cardiac patients |
| Sample age | From abstract - methods or results section |
| Sample gender | Male |
|  | Female |
|  | Both |
| Setting | Community |
|  | Healthcare facility |
|  | Educational institute |
|  | Other |
| Country study conducted | See drop down list in database (may choose more than one) |

**Measurement Data Fields**

| **Data fields for measure** | **Entry options** |
| --- | --- |
| Measure name | Full name of measure |
| Is it a new tool? | Novel (new) |
|  | Existing |
| Number of items | Number of questions in the instrument |
| Training | Required |
|  | Not required |
| Time to complete measurement | Less than 5 minutes |
|  | 6 -30 minutes |
|  | 31-60 minutes |
|  | Over 60 minutes |
| Access (availability) to measure | Free – found in the article or link from article |
|  | Pay – as indicated in the article / link |
|  | Contact author - if measure is not freely available then the author should be contacted. |
| Contact Info | Email of author or link to article /site |
| Name of Arabic Measure | If available |
| Description | As found in the article or a search for information on the measure is conducted |
| Acronym (abbreviation of name) | If available |
| Area of assessment (domain) | See drop down list in database (may choose more than one) |
| Condition (diagnosis) | See drop down list in database (may choose more than one) |
| Age group | Child/ adolescent (0-19 years) |
|  | Adult (over 19 years) |
|  | Other (a combination of child/adult category) |
