# Supplementary material for: Development of the Arabic Health Measures database: a bibliometric analysis of Arabic health-related measures
Source: Health Res Policy Syst. 2022 Aug 9;20:87. doi: 10.1186/s12961-022-00890-7 (PMC9361637; doi:10.1186/s12961-022-00890-7)
Supplement: Supplementary file 2 — Additional file 2. Use case diagram. [file 12961_2022_890_MOESM2_ESM.docx]

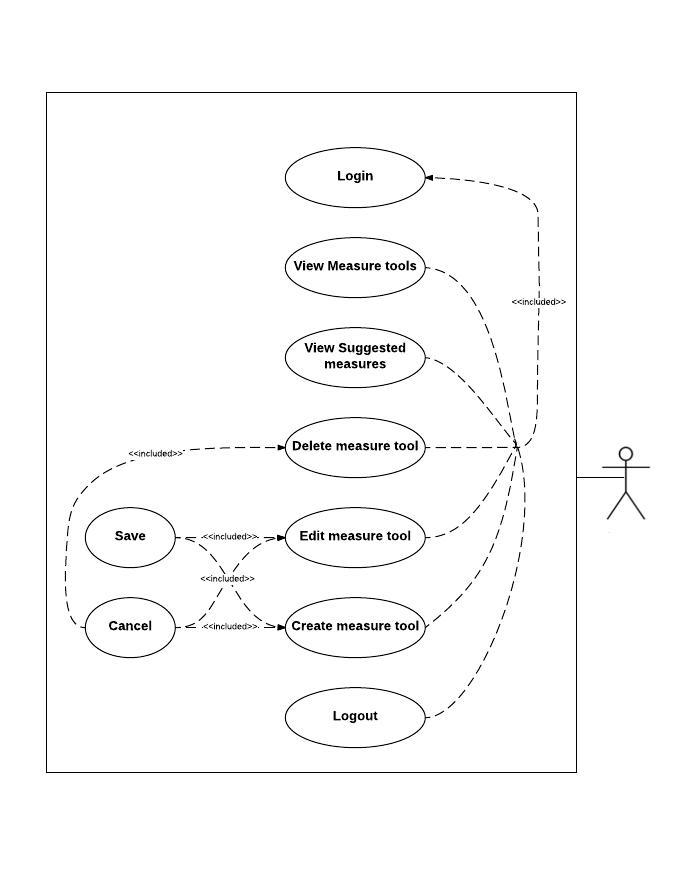

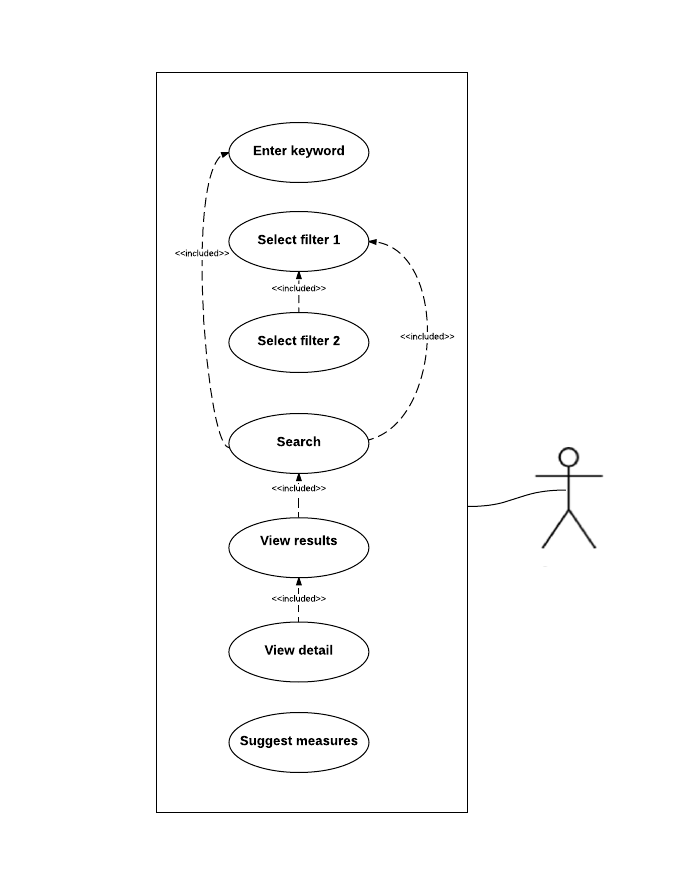


**Use case diagram (Admin) Use case diagram (Public)**

**AHM database Home page**

**AHM database search results page**
